# Mechanisms Mediating Environmental Chemical-Induced Endocrine Disruption in the Adrenal Gland

**DOI:** 10.3389/fendo.2015.00029

**Published:** 2015-03-04

**Authors:** Daniel B. Martinez-Arguelles, Vassilios Papadopoulos

**Affiliations:** ^1^Department of Medicine, Research Institute of the McGill University Health Centre, McGill University, Montreal, QC, Canada; ^2^Department of Biochemistry, Research Institute of the McGill University Health Centre, McGill University, Montreal, QC, Canada; ^3^Department of Pharmacology and Therapeutics, Research Institute of the McGill University Health Centre, McGill University, Montreal, QC, Canada

**Keywords:** reduced representation bisulfite sequencing, epigenetics, adrenal gland, phthalates, DEHP, inflammation, peroxisome proliferator-activated receptor, endocrine disruptors

## Abstract

Humans are continuously exposed to hundreds of man-made chemicals that pollute the environment in addition to multiple therapeutic drug treatments administered throughout life. Some of these chemicals, known as endocrine disruptors (EDs), mimic endogenous signals, thereby altering gene expression, influencing development, and promoting disease. Although EDs are eventually removed from the market or replaced with safer alternatives, new evidence suggests that early-life exposure leaves a fingerprint on the epigenome, which may increase the risk of disease later in life. Epigenetic changes occurring in early life in response to environmental toxicants have been shown to affect behavior, increase cancer risk, and modify the physiology of the cardiovascular system. Thus, exposure to an ED or combination of EDs may represent a first hit to the epigenome. Only limited information is available regarding the effect of ED exposure on adrenal function. The adrenal gland controls the stress response, blood pressure, and electrolyte homeostasis. This endocrine organ therefore has an important role in physiology and is a sensitive target of EDs. We review herein the effect of ED exposure on the adrenal gland with particular focus on *in utero* exposure to the plasticizer di(2-ethylehyl) phthalate. We discuss the challenges associated with identifying the mechanism mediating the epigenetic origins of disease and availability of biomarkers that may identify individual or population risks.

## Introduction

The safety of chemical compounds that pollute our environment is a topic of wide concern. Some of these pollutants have structures that mimic endogenous ligands and therefore interfere with hormone biosynthesis and metabolism, resulting in altered endocrine homeostasis (1). Identification of these chemicals, known as endocrine disruptors (EDs), is hindered because of the lack of screening tools (2). The ability to identify potential endocrine-disrupting effects is critical because an estimated 1000 new chemicals are introduced each year with only minimal pre-market safety testing. One of the most well-known EDs is thalidomide, which cause limb malformations, eye and heart digenesis, and other defects in organogenesis in babies born to women who take this drug during pregnancy (3). These striking abnormalities have led to public outrage, implementation of new regulations for drug use, and removal of thalidomide from the market. However, for most EDs, high doses are required before a clear phenotype can be identified, which has resulted in controversy about the risks of life-long exposure to low doses of EDs (4). One example is the plasticizer bisphenol A (BPA). This chemical was first introduced in the market in the 1920s, and its production has reached 2.2 million tons in recent years. Despite overwhelming evidence of its deleterious effects, there is still debate about safe exposure levels of BPA. Moreover, identifying the risk associated with a single ED is complex because we are exposed to low doses of several hundred chemicals starting at conception. These life-long environmental exposures, which include lifestyle factors, are collectively known as the exposome and will need to be taken into consideration for comprehensive risk assessments in the future (5). To study these complex interactions, tools based on omic technologies are currently being developed (6), along with *in silico* methods to predict ED activity (7).

Despite the important role of the adrenal gland in cardiovascular physiology and stress as well as evidence that steroidogenic enzymes are affected by EDs, research has been lagging. This problem was acknowledged by Hinson et al. (8) and Sanderson et al. (9) in their 2006 reviews of the effects of EDs on the adrenal gland. Since then, few studies have described cellular mechanisms underlying the effects of environmental pollutants on adrenal gland function.

Fetal development is a period of high plasticity that may be negatively influenced by exposure to environmental pollutants, resulting in disease later in life (10). In particular, the plasticizer di(2-ethylhexyl) phthalate (DEHP) is widely used in industry as an additive to polyvinyl chloride products. Exposure to DEHP has been shown to alter gonadal steroidogenesis, and levels of DEHP or its metabolites are positively correlated with human disease. Here, we will focus on the effects of *in utero* DEHP exposure on adult adrenal steroid biosynthesis, examining recent evidence for mechanisms that appear to mediate the long-term effects of fetal exposure to DEHP.

## The Testes and Adrenal Gland Share a Common Developmental Origin

The adrenal cortex and gonads originate from a common structure, the adrenogenital primordium, which is derived from the intermediate mesoderm (11). In the rat, the adrenogenital primordium divides into two distinct cell populations at gestational day (GD) 11.5, which gives rise to the adrenal cortex and the bipotential gonad [reviewed in Ref. (12, 13)].

In the rat testis, Leydig cells develop in two waves of proliferation and differentiation; these cells are thought to arise from mesenchymal precursors (14). Fetal-type Leydig cells differentiate at GD 14 and reach peak testosterone production at GD 19, driving primary sex organ development. Thereafter, fetal-type Leydig cells decrease testosterone production and disappear by postnatal day (PND) 10. Testosterone levels remain low until puberty, when adult-type Leydig cells initiate differentiation and testosterone biosynthesis, which continues for the remainder of life.

The fetal adrenal gland consists of two distinct zones: an inner cell layer known as the fetal zone, and an outer layer known as the definitive zone (15). During GD 12–14, the fetal adrenal undergoes encapsulation, which coincides with the migration of cells that originate at the neural crest. These neural cells will become chromaffin cells in the medulla and release epinephrine under sympathetic regulation (16). From PND 1 to 7, the adrenal gland undergoes zonation, which is characterized by compaction of the chromaffin cells into the medulla and regression of the fetal zone. This is followed by tissue remodeling of the definitive zone to form the zona glomerulosa (ZG), zona fasciculata (ZF), and zona reticularis (ZR). Around PND 11–21, a transient layer of undifferentiated cells called the X-zone develops between the ZF and ZR (13). This zone disappears at maturity in males and regresses after the first pregnancy in females. Steroid production is confined to the adrenal cortex, where in humans the ZG produces aldosterone, the ZF produces cortisol, and the ZR produces dehydroepiandrosterone (DHEA) and dehydroepiandrosterone sulfate (DHEAS).

Adrenal organogenesis is regulated by several key genes. The *WT1* gene, which encodes the Wilms tumor 1 protein, is essential for the genesis of the urogenital ridge. Mice lacking WT-1 function show agenesis of the kidneys, adrenal glands, and gonads (17). Other genes specific to adrenal cortex development are *SF-1* (*NR5A1*) and *DAX-1* (*NR0B1*). SF-1 knock-out mice also exhibit adrenal agenesis (18). Postnatally, SF-1 is expressed in the definitive zones of the adrenal cortex, interacting with Dax-1 to maintain adequate steroidogenic function (19). Dax-1 knock-out mice show persistency of the X-zone after puberty and are infertile (20). Although Dax-1 knock-out mice do not exhibit adrenal insufficiency, experiments involving SF-1 and Dax-1 knock-out mice revealed that these two genes are essential for correct adrenal function (21). Other genes such as *NR4A1* (*Nurr77* or *NGFI*-B) are involved in adrenal zonation and expression of key adrenal steroidogenic enzymes (22).

## Adrenal Gland Steroidogenesis

**Figure 1:**
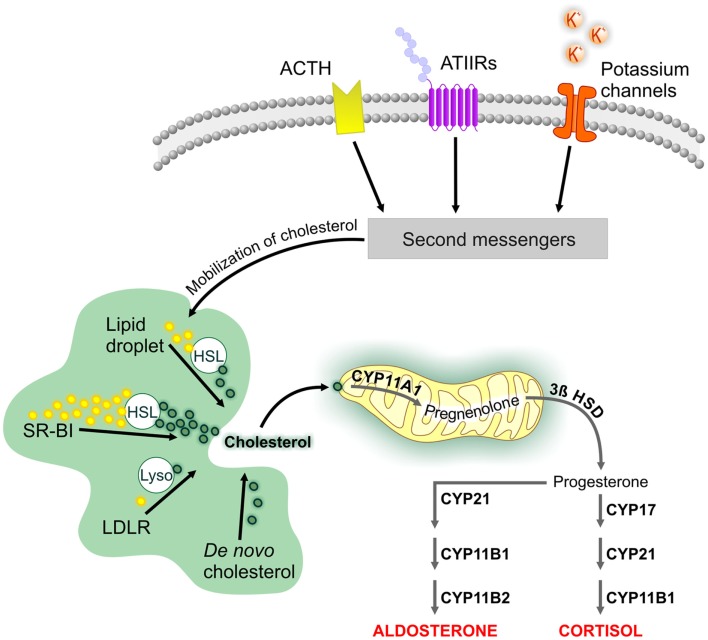
**Cellular mechanisms involved in the biosynthesis of adrenal steroids**. The cellular mechanisms that underlie adrenal steroidogenesis are initiated by activation of the adrenocorticotropic hormone (ACTH) receptor and angiotensin II type I (ATIIR) receptor by ACTH and angiotensin II, respectively. Steroidogenesis is also initiated by changes in serum potassium levels through the specific expression of potassium channels in the zona glomerulosa. This is followed by the activation of second messengers, which initiate cholesterol mobilization. Cholesterol can be obtained from LDL and HDL lipid carriers, lipid droplets, or *de novo* synthesis. Unesterified cholesterol is transported to the mitochondria, where cleavage by CYP11A1 is the first step of steroidogenesis. Tissue-specific expression in the various zones of the adrenal cortex leads to aldosterone and cortisol biosynthesis. Abbreviations: 3β HSD, 3β-hydroxysteroid dehydrogenase; HSL, hormone-sensitive lipase; LDLR, low-density lipoprotein receptor; SR-BI, scavenger receptor class BI.

The cellular mechanisms that initiate steroid biosynthesis are specific to the adrenal cortex layer (Figure 1). In the ZG, aldosterone production is stimulated by numerous molecules; however, angiotensin II, potassium, and adrenocorticotropic hormone (ACTH) have the most physiological relevance (23). The ZG expresses the angiotensin II type I receptor, which is part of the renin–angiotensin system that maintains blood pressure and water balance. The ZG also strongly expresses TWIK-related acid-sensitive potassium channel (TASK) 1 and TASK3, two-pore domain potassium channels that detect changes in circulating potassium levels (24). TASK1, which is also found in the ZF and ZR, appears to be involved in adrenal development since its deletion results in hyperaldosteronism and altered adrenal zonation (25–27). The ACTH receptor is expressed throughout the adrenal cortex and mediates the biosynthesis of glucocorticoids and androgens in the ZF and ZR (28). Ligand-mediated activation of these receptors and changes in extracellular potassium levels stimulate the release of second messengers that initiate cholesterol mobilization and prime the mitochondria for steroidogenesis.

Cholesterol serves as the substrate for all steroid hormones [reviewed in Ref. (29)]. Cholesterol for steroid biosynthesis can be obtained from extracellular or intracellular sources or can be synthesized *de novo*. The preferred sources of cholesterol are tissue-specific; here we will focus on pathways preferred by the adrenal gland. Extracellular cholesterol can be imported into the cell by the low-density lipoprotein (LDL) or scavenger receptor class BI (SR-BI) pathways (30). The LDL pathway internalizes lipoproteins containing apolipoprotein B or E and releases unesterified cholesterol after hydrolysis by lysosomal acid lipase. The free cholesterol can then be used for steroidogenesis or re-esterified and stored in lipid droplets by acyl CoA:cholesterol acyltransferase (31). In contrast to its role in the Leydig cell, the LDL pathway is a minor source of cholesterol for steroidogenesis in the adrenal gland. The SR-BI pathway mediates the uptake of high-density lipoprotein (HDL)-bound lipids (32) and is the main source of cholesterol for adrenal steroid biosynthesis. Esterified cholesterol delivered by this pathway is de-esterified by the hydrolase action of the hormone-sensitive lipase (HSL), encoded by the *LIPE* gene (33). HSL is responsible for 90% of the cholesterol ester hydrolysis activity in the adrenal gland (34, 35). Lipid droplets also contain esterified cholesterol and require HSL function to provide free cholesterol for steroidogenesis. *De novo* cholesterol synthesis starts by combining acetyl CoA and acetoacetyl CoA precursors into 3-hydroxy-3-methylglutaryl-coenzyme A (HMG-CoA) in a reaction catalyzed by HMG-CoA synthase. This is followed by the rate-limiting step in *de novo* cholesterol biosynthesis, the production of mevalonate catalyzed by the enzyme HMG-CoA reductase, which is the target of statin drugs (36). Although *de novo* cholesterol biosynthesis may help maintain adrenal gland steroidogenesis, studies have shown that lipoprotein-bound cholesterol is essential for normal adrenal steroidogenesis (37).

Transport of cholesterol into the mitochondria is aided by the steroidogenic acute regulatory protein (StAR) and a protein complex known as the transduceosome (38). Once inside the mitochondrion, cholesterol is cleaved into pregnenolone by the cholesterol side-chain cleavage enzyme (encoded by the *CYP11A1* gene) to initiate steroidogenesis. Zone-specific expression of steroidogenic enzymes results in biosynthesis of aldosterone in the ZG, cortisol in the ZF, and DHEA and DHEAS in the ZR. In rodents, the lack of adrenal *Cyp17a1* expression results in the production of corticosterone and the absence of DHEA and DHEAS (39).

## The Plasticizer DEHP Pollutes Our Environment

Di(2-ethylhexyl) phthalate (DEHP) is used in industry to increase the malleability of polyvinyl chloride and as an additive to consumer products such as cosmetics (40). DEHP can comprise up to 40% of the dry weight of polyvinyl chloride products, and because it is not permanently bound to the polyvinyl chloride matrix, it is eventually released into the environment. Because of the widespread use of DEHP, it has become a ubiquitous pollutant, contaminating our food sources and resulting in life-long exposure that ranges from 1.7 to 52.1 μg/kg/day (41–44). Food is the main source of phthalates such as DEHP, which are present in particularly high levels in fatty foods (45). Additional routes of exposure occur through dermal contact, house dust (46), and medical interventions. For example, high phthalate levels in medical equipment, such as blood (47, 48) and parenteral nutrition (49) bags and other tubing equipment, account for some of the highest exposures recorded (50). Phthalates are also used as drug excipients (51), and their use correlates with high urinary levels of its metabolites (52, 53). DEHP is rapidly metabolized, and despite its lipophilic properties, neither DEHP nor its metabolites are stored in the body (54–58). DEHP and its metabolites have been detected primarily in bodily fluids, and their presence in amniotic fluid (59) and umbilical cord blood (60) indicate direct fetal contact with the plasticizer. Exposure continues through the newborn’s environment (61), baby formula, or breast milk (62). Concerns have been raised about the risks of phthalates in newborns and infants, because they have the highest levels of exposure, whether from the environment or perinatal medical interventions (63–65).

## Fetal Development is a Sensitive Window for Endocrine Disruption in the Adult

Rat models have been extensively used to assess the endocrine-disrupting effects of phthalates. However, the windows of treatment and endpoints vary considerably across studies, making the data difficult to compare [reviewed in Ref. (66)]. In general, the results indicate that even low-dose exposure during fetal development and the neonatal period results in long-lasting phenotypes. The prepubertal period is another time of increased sensitivity to phthalates, and these compounds can cause reproductive and endocrine abnormalities. In the adult, sensitivity to phthalates is decreased, and endocrine disruption is induced only by exposure approaching the gram per kilogram per day range (67, 68). Taken together, these findings suggest that the most sensitive targets of phthalates appear during early development of the endocrine system. Moreover, mixtures of phthalates, which are more representative of human exposure, decrease the threshold for endocrine disruption (69, 70).

To evaluate long-term outcomes of fetal DEHP exposure, we treated (by gavage) pregnant Sprague–Dawley rats from GD 14 of their offspring until birth (71). During the acute exposure, DEHP decreased fetal testosterone levels and expression of steroidogenic enzymes involved in androgen biosynthesis. Immunostaining of fetal testis sections with nestin, a marker of fetal-type Leydig cells, revealed delayed Leydig cell development (72). Following birth, DEHP is rapidly metabolized in both dam and offspring, and they become phthalate-free soon after. However, *in utero* exposure to DEHP led to decreased circulating testosterone levels in the adult rat, but steroidogenic enzymes were not affected and Leydig cell numbers were near normal or even increased in adults (71). These data suggest the existence of mechanisms independent of the classic steroidogenic pathway that mediate long-term endocrine-disrupting effects of phthalates. Since fetal- and adult-type Leydig cells have different precursors, this finding also indicates that DEHP affects the development of adult-type Leydig cell precursors or the function of another organ that interacts with the testes to modulate testosterone production in adults.

We then studied nuclear receptors in an attempt to identify an alternative pathway that mediates the long-term effects of DEHP in the adult, and found that the mineralocorticoid receptor (MR), encoded by *Nr3c2*, is a novel target of DEHP in Leydig cells (72). *In utero* DEHP exposure leads to a decrease in MR expression similar to the decrease in testosterone level. MR stimulates androgen biosynthesis and potentiates the function of luteinizing hormone in purified adult Leydig cells (73). We also found that circulating levels of aldosterone, the endogenous ligand of MR, were also decreased in a manner similar to that of testosterone and MR (74). Taken together, these findings support the idea that Leydig cells are under-stimulated after DEHP exposure and identify the adrenal cortex as an additional target of DEHP. We therefore shifted our focus to the adrenal cortex, where we sought to identify the mechanisms by which DEHP affects steroidogenesis. The altered adult steroidogenic profile suggested that *in utero* exposure chosen presented DEHP with a sensitive target that has a critical role during fetal development. We hypothesized that this unknown target is likely shared by the testes and adrenal cortex because of their common mesodermal origin.

## *In utero* DEHP Exposure Alters Adult Adrenal Steroidogenesis and Cardiovascular Physiology

Our previous study showed that *in utero* DEHP exposure decreases testosterone and aldosterone levels in adult male offspring at doses starting at 100 mg/kg/day (74). Thus, exposure to 100 mg/kg/day DEHP appears to be the threshold for adult endocrine disruption following an *in utero* exposure.

The main challenge associated with identifying the molecular mechanisms underlying the antisteroidogenic effects of DEHP is the long duration of the experiments. This limits the use of certain techniques, such as primary cell culture and the number of animals and doses used to study the physiology of the DEHP-exposed animals. Instead, we and others have relied on global gene expression assays and other omic techniques to obtain a snapshot of the pathways dysregulated at specific doses and time points. We selected 300 mg/kg/day as the initial dose to evaluate the effects of DEHP. This dose decreases testosterone and aldosterone levels to approximately half of normal without causing major morphological abnormalities (71, 72).

In male offspring, aldosterone levels were not affected by *in utero* DEHP exposure at PND 21; it was only at PND 60 that aldosterone levels were decreased. The effects of endocrine disruption were still observed in the elderly rat (PND 200), where mineralocorticoid levels remained reduced (75). Aldosterone controls water and electrolyte balance by acting on the ion pumps of distal tubules and collecting ducts of the kidney to absorb sodium and excrete potassium. The reabsorption of sodium carries water, which increases intravascular volume and cardiac preload, thereby increasing blood pressure. The latter mechanism is part of the renin–angiotensin–aldosterone system, which regulates blood pressure in the short- and long-term.

To better understand the effects of DEHP on the cardiovascular system, we surgically implanted a transducer into the aorta to continuously monitor blood pressure in the unrestrained rats. The data showed that systemic blood pressure was decreased approximately 5 mmHg during the night, a period of higher activity in rats (75). Blood pressure was further decreased in older rats (PND 200) by a low-sodium diet (0.01% NaCl), which lowered diastolic blood pressure, but the DEHP-induced changes were abolished by a high-sodium diet (8% NaCl). Moreover, the low-sodium diet raised aldosterone levels 12-fold; the DEHP-treated and control rats had similar aldosterone levels, demonstrating that DEHP-exposed adrenal glands retained the capacity to maximally produce steroids and suggesting that the mechanisms regulating basal aldosterone biosynthesis were the primary target of DEHP. The decreases in blood pressure were not observed in young adult rats (PND 60), indicating that additional factors are involved in this effect on cardiovascular function. Whether heart function, vascular endothelia, or feedback mechanisms involved in regulating blood pressure are also targeted by *in utero* DEHP exposure remains unclear. However, *in vitro* models of acute DEHP exposure in cardiomyocytes have suggested a link to arrhythmias (76) and altered cardiomyocyte metabolism (77).

The decrease in blood pressure underpins the effect of DEHP on endocrine function and cardiovascular physiology, and suggests that DEHP may even have cardioprotective effects. However, we recently found evidence to the contrary. DEHP-exposed rats exhibit chronic low-grade systemic inflammation and macrophage infiltration into adipose tissue (78). Similar observations were observed in the epithelial cell line A549, which increases production of IL-6 and IL-8 in response to long-chain phthalates (79). In addition, acute *ex vivo* exposure to DEHP increases the inflammatory marker CD11b in human and rat neutrophils (80). In humans, high urinary levels of phthalate monoesters are associated with elevated levels of the inflammatory markers C-reactive protein and gamma-glutamyltransferase (81). These data indicate that DEHP induces inflammation during *in utero* and acute exposures. In addition, high urinary levels of phthalates are associated with a subclinical increase in blood pressure in children and adolescents (82). Although DEHP decreased blood pressure in our model, it is important to note that the exposure window was limited to the fetal stage. It still needs to be demined if a different exposure window or a life-long exposure to DEHP targets additional cardiovascular components that may negatively impact blood pressure.

## *In utero* DEHP-Induced Endocrine Disruption is Confined to the Adrenal Gland

After *in utero* DEHP exposure, circulating levels of aldosterone in rats are normal at the time of weaning (PND 21). Thus, DEHP does not appear to target adrenal endocrine function until the young adult stage (PND 60) (74). Since corticosterone levels were not affected at either time point, fetal exposure resulted in altered ZG development.

To determine whether the effects of DEHP on aldosterone biosynthesis were confined to the adrenal gland, we measured circulating levels of aldosterone secretagogues. Levels of potassium, angiotensin II, and ACTH were not altered in DEHP-treated rats, supporting the idea that the DEHP effects were confined to the adrenal gland. The decreased aldosterone levels were in the low-normal range, suggesting that feedback mechanisms prevent an electrolyte imbalance that would pose an immediate threat to life.

Taken together, our research findings suggest that *in utero* DEHP exposure exerts long-term effects on the expression of genes involved in regulatory functions of the adrenal gland. We will group the genetic findings into (i) cellular mechanisms that control aldosterone, (ii) cholesterol and lipid metabolism, and (iii) epigenetic mechanisms mediating endocrine disruption.

## *In utero* DEHP Exposure Affects Mechanisms Regulating Aldosterone Secretion

In search of mechanisms underlying altered aldosterone production in the adrenal gland, we found that angiotensin II receptor expression was decreased in rats exposed to DEHP *in utero* (74). This finding suggests under stimulation of the ZG to initiate aldosterone production. However, a recent transcriptomic analysis of whole adrenal glands suggested that the mechanism behind angiotensin II receptor downregulation is more complex than initially thought (83). We analyzed global gene expression at two time points to identify pathways that reflected long-term effects of DEHP exposure. Adrenal glands from male offspring exposed *in utero* to DEHP (100 or 300 mg/kg/day) were collected at PND 21 and PND 60. These time points were selected to compare the effects of DEHP during a period in which aldosterone production was not affected (PND 21) versus a period in which aldosterone production had previously been shown to be decreased (PND 60). The results showed that although DEHP deregulated a similar number of genes at the two time points, there were few genes in common among the doses and time points, suggesting that DEHP affects adrenal gland gene expression in a dose- and time-specific manner.

We then evaluated the angiotensin II and potassium pathways, comparing genes affected by DEHP in these pathways against a list of genes known to be upregulated by aldosterone secretagogues. This gene profile was obtained by isolating glomerulosa cells from adult rats stimulated with 100 mM angiotensin II or 16 mM potassium for 2 h (84). Our analysis showed that DEHP increases the expression of genes regulated by the angiotensin II and potassium pathways at PND 60 but not at PND 21. These findings suggest that the adrenal gland was chronically activated to produce steroids, perhaps to counteract the endocrine-disrupting effects of DEHP. Angiotensin II receptor expression is dynamically regulated and it is unclear whether the downregulation of the angiotensin II receptor is in response to chronic stimulation of the adrenal glands by DEHP.

These data also suggest that the potassium pathway plays an important role in DEHP-induced endocrine disruption. We found that genes encoding potassium channels *Kcnk5*, *Kcnn2*, and *Kctd14* were affected by DEHP in the adult rat. However, expression of these potassium channels was altered only at PND 60, suggesting that they mediate the chronic activation of the adrenal gland. Recently, decreased *KCNK5* (TASK-2) expression was identified as a hallmark of aldosterone-producing adenomas, and its transfection into human adrenal cell lines H295R and HAC15 cells resulted in increased aldosterone production (85). Further studies are needed to determine whether DEHP directly targets any of these potassium channels or whether these channels are part of a feedback mechanism that counteracts the effects of DEHP.

## Cholesterol Metabolism is a Sensitive Target of Plasticizers

Cholesterol import into the mitochondria is the rate-limiting step in steroid biosynthesis (38). Thus, interference in mitochondrial cholesterol transport is likely to alter steroid hormone levels. Phthalates appear to affect cholesterol metabolism in MA-10 mouse Leydig tumor cells, which show decreased expression of enzymes involved in testosterone production but retain the ability to produce progesterone and its sensitivity to hormonal stimulation (86). Treatment of MA-10 cells with mono-ethylhexyl phthalate (MEHP) for 24 h resulted in altered mitochondrial morphology and an increased number of lipid droplets starting at 1 μM MEHP and decreased progesterone levels at 30 μM MEHP (87). Similar findings were reported in 20-day-old prepubertal rats treated with DEHP (500 mg/kg/day administered by gavage for 10 days), which exhibited decreased serum progesterone and estradiol levels (88). *Ex vivo* cultures of granulosa cells isolated from these animals showed a decreased ability to produce progesterone under basal and stimulated conditions. Moreover, the use of 22-hydroxycholesterol, a cholesterol derivative that bypasses cholesterol transport machinery and diffuses freely into the mitochondria, restored steroid production in these rats, providing additional evidence that MEHP interferes with mitochondrial cholesterol transport (88). Together, these data suggest that acute exposure to DEHP/MEHP targets organelles involved in cholesterol storage and mechanisms involved in cholesterol import into mitochondria.

In our model, DEHP did not affect total circulating levels of cholesterol, LDL, HDL, and triglycerides, consistent with the idea that *in utero* exposure specifically targets the adrenal gland. We therefore assessed the effects of *in utero* DEHP exposure on cholesterol metabolism in the adult adrenal gland. We first analyzed expression levels of genes involved in extracellular and *de novo* cholesterol synthesis pathways. We found that *Ldlr*, *Hmgcr*, *Hmgcs1*, and *Insig1* expression was upregulated by DEHP, suggesting an increase in cholesterol sources (74). This putative increase in cholesterol bioavailability was a puzzling finding since DEHP decreases aldosterone output, indicating an intracellular accumulation of cholesterol. Staining of adrenal sections with Oil Red O revealed that DEHP-induced lipid droplet accumulation was specific to the ZG, positively correlating with the doses of DEHP that decreased aldosterone levels. These data suggest the existence of an unidentified gene that is responsive to DEHP and involved in mitochondrial cholesterol transport in ZG cells.

**Figure 2:**
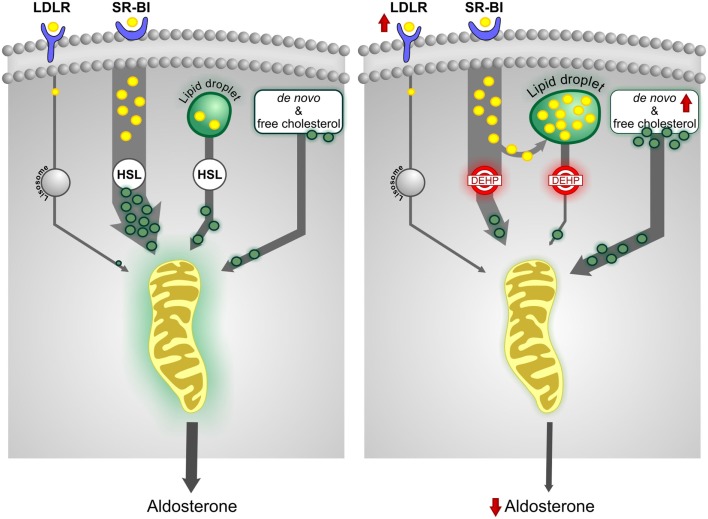
***In utero* exposure to DEHP alters cholesterol metabolism**. The scavenger receptor class BI (SR-BI) pathway, which facilitates the uptake of cholesterol from HDL, is the predominant source of cholesterol for adrenal steroidogenesis. The esterified cholesterol delivered by SR-BI and obtained from lipid droplets is de-esterified by hormone-sensitive lipase (HSL) for steroid biosynthesis. *In utero* DEHP exposure increases expression of the low-density lipoprotein receptor (LDLR) and *de novo* cholesterol synthesis pathway but decreases HSL expression. HSL function appears to be insufficient to release free cholesterol, thus decreasing the pool of free cholesterol. Some of the esterified cholesterol is shunted to lipid droplets, increasing their size. The data suggest that increased *de novo* cholesterol synthesis is critical to maintain steroidogenesis.

Analysis of global gene expression at PND 21 and PND 60 also revealed that DEHP targets the metabolism of lipids and lipoproteins in a time-specific manner. We observed changes in enzyme expression related to metabolism of fatty acids, triacylglycerol, and ketone bodies at PND 21 but not at PND 60. Conversely, the *de novo* cholesterol synthesis pathway, including the rate-limiting step and several other enzymes, was affected only at PND 60, suggesting that DEHP decreases the pool of free cholesterol below the level required to maintain adult aldosterone biosynthesis. Moreover, our pathway analysis showed that DEHP alters the expression of genes involved in intracellular cholesterol mobilization (*Lipe*, *Fabp4*, *Plin*, *MgII*, and *Prkacb*). We noted that downregulation of the gene encoding HSL (*Lipe*) matched the decreased levels of aldosterone in magnitude, suggesting a direct role in endocrine disruption (83). Consistent with our findings, experiments in *Lipe* knock-out mice revealed the accumulation of lipid droplets in the ZG and ZF (89) and adipose tissue (90). These mice had normal basal levels of corticosterone and aldosterone, suggesting that other pathways were able to supply sufficient amounts of free cholesterol (89). We therefore hypothesized that the downregulation of *Lipe* expression was involved in decreasing intracellular levels of free cholesterol, leading to activation of the *de novo* cholesterol synthesis pathway. Our results also suggested that *de novo* cholesterol synthesis was critical for steroidogenesis in adult male rats exposed *in utero* to DEHP. Figure 2 summarizes the effects of *in utero* DEHP exposure in adult adrenal cholesterol metabolism.

*In vitro* studies performed in human testes and ovaries exposed to MEHP for 72 h have identified a potential role for liver X receptor alpha (LXRα) in the upregulation of cholesterol biosynthesis genes (91). However, the role of LXRα in the long-term changes observed in our model remains unclear.

## The PPAR Pathway is a Sensitive Target of Phthalates

Pathway analysis of our global gene expression data also revealed that DEHP decreases expression of several genes downstream of the peroxisome proliferator-activated receptor (PPAR) nuclear receptors at PND 21 and PND 60 (83). At PND 21, we found that DEHP downregulated the genes encoding the retinoid X receptor (*Rxrg*), a PPAR dimerizing partner, and PPAR-gamma coactivator 1-beta (*Ppargc1b*), a coactivator of PPARγ. Despite these changes, aldosterone levels were not affected, suggesting that the PPAR pathway-related genes targeted by DEHP are not essential to maintain aldosterone levels. In untreated control rats, aldosterone levels were increased threefold at PND 60 compared to levels at PND 21 (74), correlating with a sixfold increase in *Ppara* levels at this time point, suggesting that PPARα function may be important for aldosterone biosynthesis in the adult. We quantified gene expression of the PPARs at various developmental time points, and found that DEHP decreased *Ppara* expression in adult rats at doses starting at 1 mg/kg/day. This dose is two orders of magnitude lower than the 100 mg/kg/day needed to induce endocrine disruption, demonstrating that *Ppara* is a sensitive target of DEHP. PPARs regulate lipid metabolism; therefore, PPARα may be an upstream mediator of the long-term lipid changes described in the previous section. The involvement of PPARα in the deregulation of lipid metabolism genes was also observed in fetal testes acutely exposed to DBP (92). However, it is important to note that the expression patterns of lipid-related genes after acute DBP exposure to (93) differed considerably from expression patterns observed after *in utero* DEHP exposure (83). Taken together, these data implicate the PPAR pathway as a mediator of both short- and long-term endocrine-disrupting effects of phthalates, and suggest the existence of additional mechanisms responsible for the time-specific effects.

Other members of the PPAR family have also been reported to influence adrenal steroidogenesis. PPARγ modulates aldosterone biosynthesis by modulating the expression of the gene encoding aldosterone synthase (*Cyp11b2*) in H295R (94) and HAC15 cell lines (95). These studies also demonstrated that the PPARγ agonist pioglitazone decreases aldosterone levels, even in the presence of angiotensin II stimulation. Similarly, pioglitazone was reported to suppress high aldosterone levels in a patient with primary aldosteronism (96). In our study, we found that DEHP appeared to decrease *Pparg* expression starting at 1 mg/kg/day; however, this effect was not significant (*P* = 0.09). Interestingly, *Ppard* was significantly upregulated at DEHP doses that decreased aldosterone levels. Few studies have investigated the effects of PPARδ, but studies in 3T3 cells demonstrated that PPARδ represses the ligand-induced activities of PPARα and PPARγ (97). We hypothesized that the PPAR pathway is critical for maintaining adequate aldosterone biosynthesis in the adult, and that its disruption is one of the mechanisms underlying the long-term effects of DEHP on the adrenal gland.

To identify mediators of this long-term PPAR dysfunction, we quantified the expression of selected transcription factors reported to activate PPAR promoter activity in adrenal and other tissues. We showed that DEHP significantly decreased mRNA levels of *Sp1*, *Gata6*, and *Nr1h4* and increased *Nfkb1* levels at the doses affecting aldosterone levels. Although these genes modulate several cellular functions, the role that they play in adrenal PPAR expression remains to be elucidated. Interestingly, GATA6 has been shown to mediate the expression of key steroidogenic enzymes and proteins involved in cholesterol transport to mitochondria (98). Moreover, SP-1 and PPARγ were shown to control HSL expression in the liver (99). In our model, these data suggest that GATA6 and SP-1 may mediate some of the long-term effects of DEHP. Additional research is needed to elucidate the exact role of PPARs and the transcription factors identified in our study in DEHP-induced endocrine disruption.

There is additional *in vitro* evidence suggesting that the PPAR pathway acts as a dominant regulator of steroidogenesis. Treatment of MA-10 mouse Leydig tumor cells with the PPARα agonist bezafibrate (100 μM) decreased biosynthesis of progesterone and testosterone (100). Moreover, MA-10 cells exposed to 10 or 100 μM MEHP were not able to achieve normal levels of steroid production despite stimulation with human chorionic gonadotropin (100, 101). Conversely, low doses of MEHP (0.1 μM) showed discrete increases in stimulated steroid production in MA-10 cells (102).

## Epigenetic Changes Induced by DEHP

The addition of a methyl group to cytosine in CpG dinucleotides is known as DNA methylation. This epigenetic mark influences gene expression and is one of the mechanisms responsible for cell diversity. Epigenetic changes occurring in early life in response to environmental factors such as stress or toxicants have been shown to affect behavior (103, 104), increase cancer risk (105), and modify cardiovascular physiology later in life (106). Several epigenetic mechanisms that regulate endocrine function have been identified [reviewed in Ref. (107, 108)]. The identification of epigenomic regions that interact with the environment is critical to understanding origins of disease, determining which chemicals exert long-term effects on human health, and identifying novel biomarkers for use in personalized medicine. However, research has been lagging because of the challenges associated with large-scale epigenomic screening, high cost and slow turnaround of data acquisition, and lack of established bioinformatics pipelines to process the data.

Since DEHP is rapidly metabolized, we hypothesized that changes observed in the adult after *in utero* DEHP exposure were mediated by epigenetic changes. In the testes, we identified an area of differential DNA methylation in the MR promoter (72) and time-specific DNA methylation changes in various nuclear receptor genes in adult rats exposed to DEHP *in utero*. In the adrenal gland, we searched for changes in the promoter regions of several genes affected by DEHP. We previously reported the lack of DNA methylation changes in *Agtr1a*, *Agtr1b*, and *Atrap* (74). We also analyzed the promoters of *Kcnk5* and *Kcnn2* but did not detect any DNA methylation changes (unpublished data).

We recently used reduced representation bisulfite sequencing (RRBS) to characterize DEHP-induced DNA methylation in the adrenal glands of adult male rats exposed *in utero*. RRBS uses a combination of methylation-insensitive restriction enzymes, bisulfite treatment, and next-generation sequencing to ascertain the DNA methylation levels of millions of CG dinucleotides (109). We sampled approximately 2.18 million CpGs and identified 972 differentially methylated CpGs (110). Although we expected to find differentially methylated CGs near or within promoter regions of genes upregulated or downregulated after *in utero* DEHP exposure, most of the differentially methylated CGs (40%) were found in CpG islands followed by shore/shelf regions (30%), which control gene expression. Moreover, our results showed the clustering of differentially methylated CGs throughout the genome. Some of these methylation hotspots correlated with genes affected by DEHP, within a 2.5-Mb window (110). In particular, chromosome 20p12 showed two distinct clusters of DNA methylation in a locus that contains genes that regulate immune responsiveness, including several that are part of the antigen processing and presentation pathway (*Rt1-Bb*, *Rt1-a2*, *Rt1.aa*, *Rt1-Bb*, *Hspa2*, and *Hspa1a*). We also reported that DEHP targets several genes related to the immune response in whole adipose tissue, and significantly increases serum C-reactive protein and tumor necrosis factor levels (78). In adrenals, it is unclear whether the increased expression of immune-related genes is a consequence of macrophage or another immune cell infiltration, like the one observed in adipose tissue (78). At present, it remains to be investigated whether the identified immune-related genes or the elevated inflammatory state induced by DEHP have a direct effect in aldosterone biosynthesis. Results of other studies suggest that DEHP increases inflammation in animals and humans (111, 112). Therefore, identification of this epigenetically active region may help elucidate the mechanism underlying the altered immune state induced by DEHP and serve as a biomarker of DEHP exposure.

We mapped the genomic location of differentially methylated CGs and genes deregulated by DEHP in the adrenal gland of adult rats. At the chromosome 20p12 loci, DNA methylation hotspots correlated positively with genes deregulated by DEHP. Moreover, transcriptomic data from various adrenal and non-adrenal tissues at PND 21 and PND 60 also correlated with gene deregulation at the chromosome 20p12 loci (110). Taken together, these data suggest that DNA methylation at these loci may be affected in other tissues and that these epigenetic changes regulate regional gene expression. Is important to note that RRBS covered only 9% of the genome; therefore, other CpGs may be involved in mediating the effects of DEHP.

We wondered whether these differentially methylated CGs were sensitive to environmentally relevant doses of DEHP. We therefore selected 52 CGs that exhibited the greatest DNA methylation changes, and used DNA methylation enrichment techniques to quantify their response to 1 mg/kg/day DEHP. The data showed that several loci were significantly affected at this low dose. Moreover, we identified DNA methylation changes at doses below those that affect aldosterone biosynthesis. These data together with the decreased PPARα expression at 1 mg/kg/day led us to hypothesize that exposure to low levels of DEHP acts as a first hit to the epigenome, increasing disease risk later in life.

## In Search of a Biomarker of DEHP Exposure

Despite the fact that EDs have been extensively studied, few biomarkers have been reported (113). Most studies correlating phthalate levels with human disease are based on a single urine measurement, but since DEHP is rapidly metabolized, multiple measurements are needed to accurately evaluate exposure to EDs (114). Moreover, identification of a “clean” biomarker is difficult because EDs mimic endocrine signals that may be altered in metabolic or endocrine diseases. Nevertheless, biomarkers that are easily accessible in humans are needed to confirm findings in animal models and aid in the risk assessment of EDs (115).

We therefore sought to identify biomarkers of DEHP exposure using the results of our global gene expression assays. We identified genes affected at both time points and doses in our adrenal gene expression assay. We hypothesized that these genes were the most sensitive to DEHP, and that some gene products could be measured in the serum. Our selection criteria resulted in a short list of genes, most of which were downregulated by DEHP. We quantified serum levels of aquaporin 7 (AQP7), fatty acid-binding protein 4 (FABP4), and phosphoenolpyruvate carboxykinase 1 (PCK1). Results of linear regression analysis showed that serum levels of FABP4 and PCK1 were inversely correlated to the fetal dose of DEHP (83). In particular, serum PCK1 level was increased by *in utero* exposure at 1 mg/kg/day but sharply decreased at higher DEHP levels, suggesting dose-specific effects of DEHP on PCK1 expression. Although serum levels of PCK1 and FABP4 may not necessarily be indicative of individual exposure to EDs, they may help identify exposure in high-risk populations. Further studies of these proteins and discovery of other putative biomarkers are needed to translate findings in animal models to humans (116, 117). Whether PCK1 levels remain altered by different exposure windows or different phthalates requires further study.

It is also uncertain how ED exposure may be at the origin or a participant in other public health problems such as the metabolic syndrome where obesity, abnormal glucose metabolism, and inflammation are common findings. Several studies have proposed an association between early-life exposure to an ED and increased risk of obesity later in life (118). Some of these studies have questioned whether phthalates increase obesity risk via PPARs (119). In male rats exposed to DEHP *in utero*, we identified markers of systemic and local adipose tissue inflammation (78). Moreover, the DEHP-induced downregulation of *Lipe* may also play a role in the development of metabolic syndrome, since *Lipe* knock-out mice exhibit insulin resistance in several tissues, including the liver, adipose tissue, and skeletal muscle (120). Furthermore, in humans, single nucleotide polymorphisms in the *LIPE* locus have shown gender-specific associations with plasma lipid and glucose levels (121). The discovery of novel biomarkers will aid in clarifying the relationships between ED exposures and metabolic syndrome.

## Conclusion

Numerous studies have demonstrated that *in utero* exposure to DEHP alters endocrine function in adulthood. Taken together, the results suggest that endocrine disruption is initiated at lower doses but remains latent until a second hit occurs in the form of a higher dose of DEHP or a combination of EDs. The data also show that long-term endocrine disruption is dependent on the window of exposure. Given that humans are exposed to hundreds of chemicals throughout life beginning at conception, there is a need for screening tools that can aid in the risk assessment of potential EDs.

## Conflict of Interest Statement

The authors declare that the research was conducted in the absence of any commercial or financial relationships that could be construed as a potential conflict of interest.
